# A New Cubitane Diterpenoid from the Soft Coral *Sinularia crassa*

**DOI:** 10.3390/molecules170910072

**Published:** 2012-08-24

**Authors:** Ching-Hsiao Cheng, Yun-Sheng Lin, Zhi-Hong Wen, Jui-Hsin Su

**Affiliations:** 1 Department of Neurosurgery, Chang Gung Memorial Hospital-Kaohsiung Medical Center, Kaohsiung 833, Taiwan; Email: ma4200@adm.cgmh.org.tw; 2 Department of Biological Science and Technology, Meiho University, Pingtung 912, Taiwan; Email: x00010106@meiho.edu.tw; 3 Department of Marine Biotechnology and Resources and Asia-Pacific Ocean Research Center, National Sun Yat-sen University, Kaohsiung 833, Taiwan; Email: wzh@mail.nsysu.edu.tw; 4 National Museum of Marine Biology & Aquarium, Pingtung 944, Taiwan; 5 Graduate Institute of Marine Biotechnology, National Dong Hwa University, Pingtung 944, Taiwan

**Keywords:** diterpenes, soft coral, *Sinularia crassa*

## Abstract

A new cubitane diterpenoid, crassalone A (**1**), was isolated from the marine soft coral *Sinularia crassa*. The structure was determined by extensive spectroscopic analyses. Compound **1** is not cytotoxic (IC_50_ > 20 μg/mL) toward the four human cancer cell lines tested (HL60, MDA-MB-231, HCT-116 and DLD-1).

## 1. Introduction

**Figure 1:**
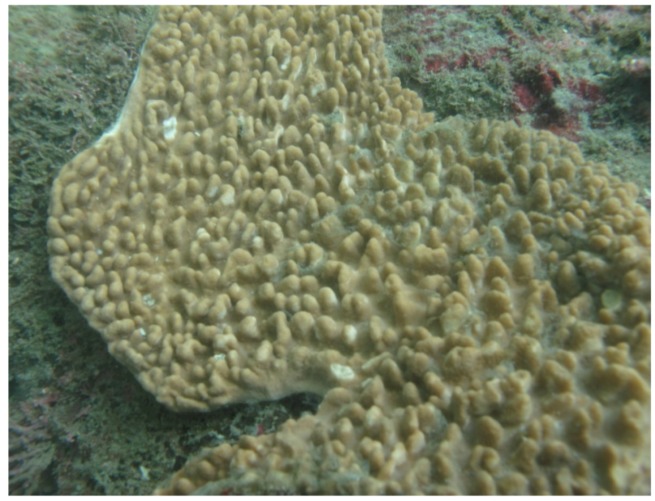
The soft coral *Sinularia crassa*.

**Figure 2:**
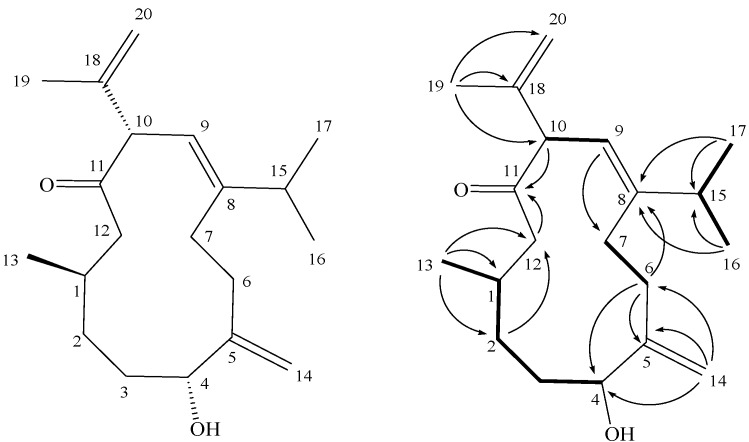
The structure of metabolite **1** and selected ^1^H-^1^H COSY (▬) and HMBC (→) correlations.

Marine soft corals of the genus *Sinularia* have attracted a great deal of attention in light of the structural diversity and wide range of biological activities of their metabolites [1]. Moreover, Formosan soft corals of the genus *Sinularia* have been shown to be rich sources of structurally unique and bioactive natural products [2,3,4,5,6]. In the investigation of secondary metabolites in marine invertebrates, the diterpenoid cubitane was first reported in 1984 as being obtained from the Caribbean gorgonian octocoral *Eunicea calyculata* [7]. In other reports, studies of the chemical constituents of octocorals have led to the isolation of various cubitane diterpenoids [7,8,9]. Recently, we have isolated two cubitane diterpenoids from the soft coral *Sinularia* *triangula* [10,11]. Our recent study of the chemical constituents on *Sinularia crassa* (Figure 1) had led to the isolation of two cembranoids [12]. In this paper, we further report the isolation of a new cubitane diterpenoid, crassalone A (**1**, Figure 2). The structure of **1** was established by extensive spectroscopic analysis, including careful examination of 2D-NMR (^1^H-^1^H COSY, HMQC, HMBC and NOESY) correlations. The cytotoxicity of compound **1** against human promyelocytic leukemia (HL60), human breast adenocarcinoma (MDA-MB-231), human colon adenocarcinoma (HCT-116) and human colorectal carcinoma (DLD-1) cell lines was also studied.

## 2. Results and Discussion

Crassalone A (**1**) was obtained as a colorless oil. The HR-ESI-MS spectrum of **1** exhibited a molecular ion peak at *m/z* 327.2298 [M+Na]^+^, and established a molecular formula C_20_H_32_O_2_, implying five degrees of unsaturation. The IR spectrum of **1** showed a broad absorption band at 3422 cm^−1^ and a strong absorption band at 1708 cm^−1^, implying the presence of hydroxy and carbonyl groups, respectively. Inspection of the ^13^C-NMR and DEPT spectral data of **1** (Table 1) in CDCl_3_, indicated the presence of 20 carbon signals of a diterpenoid. These signals were ascribable to carbons of four methyls, five sp^3^ methylenes, two sp^2^ methylenes, four sp^3^ methines (including an oxygenated carbon at δ 75.3) and one sp^2^ methine. The remaining four signals appearing in the lower field region of the spectrum are due to the quaternary carbons of thee olefinic carbons (δ 151.7, 147.9 and 142.9) and one ketone carbonyl (δ 211.3). The ^1^H-NMR spectral data revealed the presence of four olefinic methylene protons (δ 5.03, 4.90, 4.89 and 4.79, each s) and one olefinic proton (δ 5.35, d, *J* = 9.5 Hz). By interpretation of ^1^H-^1^H COSY correlations, it was possible to establish four partial structures from H-1 to H-4 and H_3_-13, from H_2_-6 to H_2_-7, from H-9 to H-10, and from H-15 to both H_3_-16 and H_3_-17 (Figure 2). These data, together with the HMBC correlations (Figure 2) from H_2_-2 to C-12, H_2_-6 to C-4, C-5 and C-8, H-9 to C-7, H-10 and H_2_-12 to C-11 (carbonyl carbon) established the connectivity within the 12-membered ring. A 1,1-disubstituted double bond attached at C-5 was confirmed by the HMBC correlations from H_2_-14 to C-4, C-5 and C-6. One methyl group attached at C-1 was confirmed by the HMBC correlations from H_3_-13 to C-1, C-2 and C-12. Furthermore, two isopropyl moieties attached at C-8 and C-10 were confirmed by the HMBC correlations from both methyl H_3_-16 and H_3_-17 to C-8 and C-15 and H_3_-19 to C-10, C-18 and C-20. Thus, **1** was found to possess one trisubstituted olefin at C-8/C-9, one ketone group at C-11, and two 1,1-disubstituted double bonds at C-5/C-14 and C-18/C-20, respectively.

**Table 1:** ^1^H and ^13^C-NMR datafor **1**.

	δ_H_ ( *J* in Hz) ^a^	δ_C_ (mult.) ^b^		δ_H_ ( *J* in Hz) ^c^	δ_C_ (mult.) ^d^
1	2.04 m	32.1 (CH)		2.10 m	32.3 (CH)
2	1.54 m; 1.16 m	34.1 (CH_2_)		1.42 m; 1.08 m	34.9 (CH_2_)
3	1.49 m	34.4 (CH_2_)		1.53 m; 1.34 m	35.2 (CH_2_)
4	4.02 brs	75.3 (CH)		3.78 dd (7.0, 5.5)	75.2 (CH)
5		151.7 (C)			152.9 (C)
6	2.39 m; 2.01 m	31.6 (CH_2_)		2.27 m; 2.07 m	32.5 (CH_2_)
7	2.31 m; 2.16 m	27.3 (CH_2_)		2.27 m; 2.00 m	28.2 (CH_2_)
8		147.9 (C)			148.0 (C)
9	5.35 d (9.5)	120.0 (CH)		5.64 d (10.0)	121.4 (CH)
10	4.03 d (9.5)	61.7 (CH)		4.00 d (10.0)	62.3 (CH)
11		211.3 (C)			209.6 (C)
12	2.45 dd (13.5, 11.0);	50.2 (CH_2_)		2.27 m;	50.7 (CH_2_)
	2.26 dd (13.5, 2.5)			2.17 dd (14.0, 2.5)	
13	0.98 d (7.0)	23.3 (CH_3_)		0.78 d (7.0)	23.8 (CH_3_)
14	5.03 s; 4.90 s	111.8 (CH_2_)		4.97 s; 4.81 s	111.5 (CH_2_)
15	2.35 m	33.0 (CH)		2.24 m	33.5 (CH)
16	1.07 d (7.0)	21.3 (CH_3_)		1.06 d (7.0)	21.8 (CH_3_)
17	1.02 d (7.0)	22.5 (CH_3_)		0.95 d (7.0)	23.1 (CH_3_)
18		142.9 (C)			143.9 (C)
19	1.72 s	21.2 (CH_3_)		1.66 s	21.5 (CH_3_)
20	4.89 s; 4.79 s	113.7 (CH_2_)		4.85 s; 4.80 s	114.0 (CH_2_)

^a^ 500 MHz in CDCl_3_; ^b^ 125 MHz in CDCl_3_; ^c^ 500 MHz in pyridine-*d*_5_; ^d^ 125 MHz in pyridine-*d*_5_.

**Figure 3:**
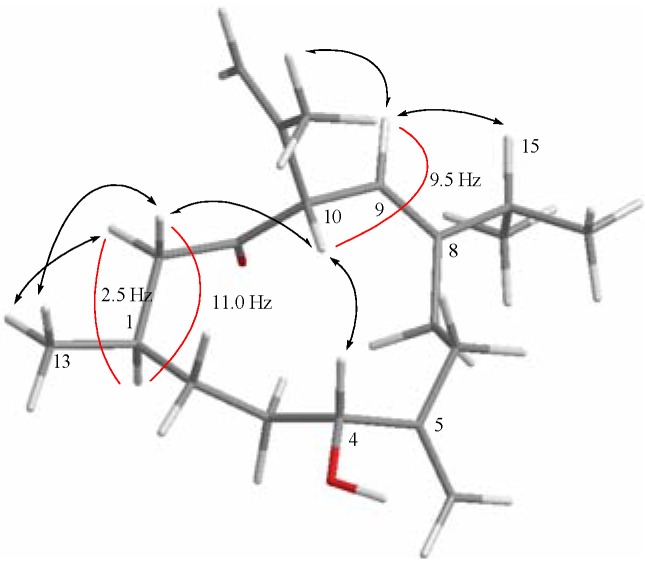
Selective NOESY correlations and coupling constants (*J*) of **1**.

**Figure 4:**
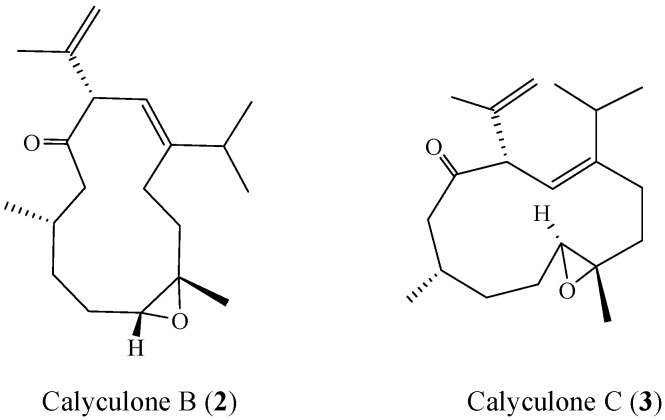
Structures of **2** and **3**.

The relative structure of **1** was elucidated by the analysis of NOE correlations, as shown in Figure 3. The NOE correlations observed between H-9 and H-15 reflected the *E* geometry of double bonds at C-8/C-9. Moreover, the *E* geometry of double bond at C-8/C-9 further established by comparison of the-NMR data of **1** in CDCl_3_ with those of two related compounds, calyculone B (**2**) and calyculone C (**3**) (Figure 4), also measured in CDCl_3_. The proton shifts of two protons [H-9 (δ_H_ = 5.35) and H-15 (δ_H_ = 2.35)] of **1**, were found to be the same as those of **2** [H-9 (δ_H_ = 5.37) and H-15 (δ_H_ = 2.28)] [7]. Also, the carbon shifts of C-9 (δ_C_ = 120.0) and C-15 (δ_C_ = 33.0) were found to be more closer to that of **2** [C-9 (δ_C_ = 119.8) and C-15 (δ_C_ = 31.7)] relative to that of **3** [C-9 (δ_C_ = 117.1) and C-15 (δ_C_ = 27.5)], too [7]. Thus, it was suggested that the double bond of **1**at C-8/C-9 should be *E* geometry. Due to the overlapping of H-4 and H-10 signals (δ 4.02−4.03 ppm) on measuring the ^1^H-NMR in CDCl_3_, we also measured the NOESY spectrum of **1** in pyridine-*d*_5_, which showed signals of both H-4 and H-10 at δ 3.78 (dd, *J* = 7.0, 5.5 Hz) and 4.00 (d, *J* = 10.0 Hz), respectively. Therefore, the NOE interaction (measured in pyridine-*d*_5_) between H-4 and H-10 suggested the 4R*,10S* configurations as depicted in Figure 3. Moreover, the consecutive NOE correlations (measured in CDCl_3_) of H_2_-12 with H-10 and H_3_-13 and not with H-1 indicate the â-orientation of H_3_-13. Finally, the *J* values of H-1/H-12a (11.0 Hz) and H-9/H-10 (9.5 Hz) revealed the *anti* geometries between the above vicinal protons, as shown in Figure 3. From the above observations, the structure of **1** was fully established.

Finally, we used a 3-(4,5-dimethylthiazol-2-yl)-2,5-diphenyl tetrazolium bromide (MTT) assay to examine the cytotoxic activities of compound **1** against four cancer cell lines, including HL60 (human promyelocytic leukemia), MDA-MB-231 [human breast adenocarcinoma (grade III)], DLD-1 (human colon adenocarcinoma) and HCT-116 (human colorectal carcinoma) cancer cells. Cells were treated with different concentrations of **1** for 72 h. The viability of the various cancer cells was not significantly decreased by 50%, even under treatment with 20 μg/mL of **1**. In addition, the IC_50_ values of compound **1** were over 20 μg/mL. The results showed that compound **1** did not possess cytotoxicity against these cancer cells.

## 3. Experimental

### 3.1. General Procedures

Optical rotation values were measured using a Jasco P-1010 digital polarimeter. IR spectra were recorded on a Varian Digilab FTS 1000 Fourier transform infrared spectrophotometer. NMR spectra were recorded on a Varian Unity INOVA 500 Fourier transform-nuclear magnetic resonance (FT-NMR) instrument at 500 MHz for ^1^H-NMR and 125 MHz for ^13^C-NMR, respectively, in CDCl_3_ and pyridine-*d*_5_. ESIMS and HESIMS data were recorded with a Bruker APEX II mass spectrometer. Gravity column chomatography was performed on silica gel (230–400 mesh, Merck, Darmstadt, Germany). Thin layer chomatography (TLC) was carried out on precoated Kieselgel 60 F254 (0.2 mm, Merck) and spots were visualized by spraying with 10% H_2_SO_4_ solution followed by heating. HPLC was performed using a system comprised of a Hitachi L-7100 pump (Tokyo, Japan) and a Rheodyne 7725 injection (Cotati, USA) port. A preparative normal phase column (Hibar 250 × 21.2 mm, Supelco, silica gel 60, 5 μm, Bellefonte, USA) was used for HPLC.

### 3.2. Animal Material

The marine soft coral *S. crassa* (Tixier-Durivault, 1945) was collected by scuba divers at a depth of around 10 m off the coast of Taitung County, Taiwan, in October 2011, and the sample was frozen immediately after collection. A voucher sample was deposited at the National Museum of Marine Biology and Aquarium, Taiwan (specimen No. 2011-1012-7).

### 3.3. Extraction and Separation

The soft coral (0.8 kg, fresh wt.) was stored frozen and then freeze dried. The freeze-dried material (350 g) was minced and extracted five times with EtOAc (1 L) for 12 h each time at room temperature. The organic extract was evaporated to yield a residue (10.5 g), which was subjected to open column chomatography on silica gel eluting with gradients of *n*-hexane (H)–EtOAc (E) and EtOAc (E)–acetone (A) gradient, to give 15 fractions: Fr-1 (eluted by *n*-hexane), Fr-2 (eluted by H–E 100:1), Fr-3 (eluted by H–E 50:1), Fr-4 (eluted by H–E 30:1), Fr-5 (eluted by H–E 20:1), Fr-6 (eluted by H–E 15:1), Fr-7 (eluted by H–E 10:1), Fr-8 (eluted by H–E 8:1), Fr-9 (eluted by H–E 5:1), Fr-10 (eluted by H–E 3:1), Fr-11 (eluted by H–E 2:1), Fr-12 (eluted by H–E 1:1), Fr-13 (eluted by EtOAc), Fr-14 (eluted by E–A 1:1) and Fr-15 (eluted by acetone). Fraction 10 was further separated by silica gel column chomatography with gradient elution (*n*-hexane–EtOAc, 5:1 to 1:1) to yield five subfractions (10A–E). Subfraction 10C was subjected to normal phase HPLC with *n*-hexane–acetone (6:1) as the eluent (flow rate 2 mL/min) to obtain compound **1** (2.0 mg, 0.019% dry wt of extract). *Crassalone A* (**1**): colorless oil; [α]_D_^25^ = −5 (*c* 0.05, CHCl_3_); UV (MeOH) λ_max_ nm: end absorption; IR (neat) ν_max_3422, 2956, 2924, 2855, 1708, 1460 and 1377 cm^−^^1^; ^1^H- and ^13^C-NMR data, see Table 1; ESIMS *m*/*z* 327 [100, (M+Na)+]; HESIMS *m*/*z* 327.2298 (calcd. for C_20_H_3__2_O_2_Na, 327.2300).

### 3.4. Cytotoxicity Testing

Cell lines were purchased from the American Type Culture Collection (ATCC). Cytotoxicity assays of compound **1** was performed using the 3-(4,5-dimethylthiazol-2-yl)-2,5-diphenyltetrazolium bromide (MTT) colorimetric method [13,14]. Doxorubicin was employed as positive control, which exhibited cytotoxic activity toward HL60, MDA-MB-231, DLD-1 and HCT-116 cancer cell lines with IC_50_ values of 0.06, 6.3, 5.7 and 0.5 µg/mL, respectively.

## 4. Conclusions

In previous studies, a series of the cubitane diterpenoids had been isolated from two gorgonian corals *Eunicea calyculata* [7,8] and *Eunicea laciniata* [9], and one soft coral *Sinularia triangula* [10,11]. The present investigation demonstrated that the metabolite **1**was inactive (IC_50_’s >20 μg/mL) towards the growth of HL60, MDA-MB-231, DLD-1 and HCT-116 cancer cells. Among the cubitane diterpenoids discovered from marine organisms, only one compound (sinutriangulin A) has been found to possess weak cytotoxicity toward the two cancer cells (CCRF-CEM and DLD-1). Therefore, compounds of this class have not been found to exhibit significant cytotoxicity from our results and previous reports.
